# Genomic and Ancestral Variation Underlies the Severity of COVID-19 Clinical Manifestation in Individuals of European Descent

**DOI:** 10.3390/life11090921

**Published:** 2021-09-05

**Authors:** Priyanka Upadhyai, Gokul Suresh, Rahul Parit, Ranajit Das

**Affiliations:** 1 Department of Medical Genetics, Kasturba Medical College, Manipal Academy of Higher Education, Manipal, Karnataka 576104, India; priyanka.u@manipal.edu (P.U.); rahulgene96@gmail.com (R.P.); 2 Yenepoya Research Centre, Yenepoya University, Mangalore, Karnataka 575018, India; gklsrsh09@gmail.com

**Keywords:** ancestral genetic variation in COVID-19 patients, genome-wide association study for COVID-19 patients, asymptomatic COVID-19 patients as control, ANE and WHG ancestries in COVID-19, multiple regression with ancestral fractions

## Abstract

The coronavirus disease (COVID-19) caused by the novel severe acute respiratory syndrome coronavirus 2 (SARS-CoV-2) is characterized by a wide spectrum of clinical phenotypes ranging from asymptomatic to symptomatic with mild or moderate presentation and severe disease. COVID-19 susceptibility, severity and recovery have demonstrated high variability worldwide. Variances in the host genetic architecture may underlie the inter-individual and population-scale differences in COVID-19 presentation. We performed a genome-wide association analysis employing the genotyping data from AncestryDNA for COVID-19 patients of European descent and used asymptomatic subjects as the control group. We identified 621 genetic variants that were significantly distinct between asymptomatic and acutely symptomatic COVID-19 patients (multiple-testing corrected *p*-value < 0.001). These variants were found to be associated with pathways governing host immunity, such as interferon, interleukin and cytokine signalling, and known COVID-19 comorbidities, such as obesity and cholesterol metabolism. Further, our ancestry analysis revealed that the asymptomatic COVID-19 patients possess discernibly higher proportions of the Ancestral North Eurasian (ANE) and Eastern Hunter-Gatherer (EHG) ancestry, which was introduced to Europe through Bell Beaker culture (Yamnaya related) and lower fractions of Western Hunter-Gatherer (WHG) ancestry, while severely symptomatic patients have higher fractions of WHG and lower ANE/EHG ancestral components, thereby delineating the likely ancestral differences between the two groups.

## 1. Introduction

Since its outbreak in December 2019 in Wuhan, China, the coronavirus disease (COVID-19) has ravaged the world, causing 217,901,675 infections and 4,523,766 deaths worldwide (https://covid19.who.int, assessed on 31 August 2021). It is caused by the novel severe acute respiratory syndrome coronavirus 2 (SARS-CoV-2) [1,2] and has a broad spectrum of clinical manifestations among patients ranging from asymptomatic, symptomatic with mild or moderate respiratory disease, severe alveolar damage, pneumonia and respiratory failure [3,4,5]. More than 35% of infected individuals display neurological symptoms ranging from headache, loss of smell (anosmia), loss of taste (ageusia), dizziness and cerebrovascular disease [6]. Gastrointestinal, cardiac, kidney and vascular abnormalities are also observed in COVID-19 patients [7]. Overall mortality upon hospitalization is 15–20% but increases to ~40% for patients requiring intensive care [8]. Long-term effects of COVID-19 infection may involve significant sequelae, especially for grievously affected individuals, including microstructural and functional brain anomalies in more than 50% of cases [9].

Strikingly COVID-19 susceptibility, severity and recovery have shown high variability worldwide. Older adults ≥ 60 years [10] and those with pre-existing comorbidities (e.g., hypertension, cardiovascular disease and diabetes) [11,12,13] or habits (e.g., smoking) [14] are likely to be more vulnerable to severe SARS-CoV-2 infection. However, poor disease prognosis has also been observed in middle-aged individuals (40–59 years) with no apparent underlying health conditions [15]. Differences in disease prognosis and outcomes in worldwide populations may be attributed to variable degrees of testing and screening, different thresholds for hospitalization, differences in availability of good quality clinical care and compliance to public health measures for containing infection spread. In addition, existing studies suggest that variability in the host genetic constitution may modulate the inter-individual and population-scale differences in COVID-19 severity and clinical outcomes. These include the identification of two genetic susceptibility loci associated with respiratory failure in COVID-19, including the ABO locus in chromosome 9 and a gene cluster at chromosome 3 [16], population-specific variation of the coding variants of *Angiotensin-converting enzyme 2* (*ACE2*), the SARS-CoV-2 receptor for host cell entry [17,18,19] and that of the COVID-19 risk haplotype originating from Neanderthal genomes [20].

So far, results from several genome-wide association studies (GWAS) have identified potential genomic loci associated with the severity of COVID-19. The GWAS conducted by the Genetics Of Mortality In Critical Care (GenOMICC) assessed critically ill COVID-19 patients from UK intensive care units (ICUs) and identified *2′-5′-Oligoadenylate Synthetase* (*OAS*) gene cluster on chromosome 12 (*OAS1*, *OAS2* and *OAS3*), a single nucleotide variant (SNV) each in *Tyrosine Kinase 2* (*TYK2*) and *Dipeptidyl Peptidase 9* (*DPP9*) genes on chromosome 19 and a single nucleotide polymorphism (SNP) in the interferon receptor gene *Interferon Alpha and Beta receptor subunit 2* (*IFNAR2*) on chromosome 21. While *DPP9* and *TYK2* are thought to be associated with host-driven inflammatory lung injury linked to life-threatening COVID-19, *IFNAR2* and *OAS* genes have been associated with innate antiviral defences [21]. Consistent with the findings of Ellinghaus et al. (2020) [16], Shelton et al. (2021) reported a strong association between *ABO* locus on chromosome 9 and another gene-rich locus on chromosome 3 that includes *Leucine zipper transcription factor-like 1* (*LZTFL1*) and *Solute Carrier Family 6 Member 20* (*SLC6A20*) with the acuteness of COVID-19 using data from more than a billion participants obtained from 23andMe [22]. An association study based on ~50,000 COVID-19 patients further supported the association of genetic variants in *ABO*, *TYK2*, *DPP9*, *IFNAR2*, *SLC6A20* and *Protein Phosphatase 1 Regulatory Subunit 15A* (*PPP1R15A*) with the severity of COVID-19 [23]. Finally, the AncestryDNA COVID-19 host genetic study identified COVID-19 genetic associations with *SLC6A20*, *LZTFL1* variants on the chromosome, *ABO* locus on chromosome 9 and a novel association on chromosome 11 that includes *Polypeptide N-Acetylgalactosaminyltransferase 18* (*GALNT18*) [24].

While the existing GWAS reports have been extremely insightful, their choice of individuals selected as controls might not be optimal. Largely, these studies have recruited healthy individuals who had tested negative for COVID-19 by reverse transcription PCR (RT-PCR) test in the control group or employed population controls [16,21,22,23,24]. Accordingly, the individuals considered in the control set had no known history of COVID-19 at the time of their recruitment, but the possibility of subsequent SARS-CoV-2 infections and their severity in them remained unaccounted. Consequently, we argue that these individuals are likely not suitable controls for predicting the underlying genetic variants associated with severe COVID-19.

Here, we utilized the genotyping dataset generated by the AncestryDNA COVID-19 host genetic study corresponding to 11,759 healthy control individuals who tested negative for COVID-19 using swab tests and 3241 COVID-19 positive individuals, encompassing 675,370 SNVs [24]. This dataset comprises ~75% of individuals with European ancestry. We categorized the SARS-CoV-2 infected patient group into five categories denoting the acuteness of manifestation, namely, asymptomatic, mild, moderate, severe and unknown, based on self-reported responses that were collected as described before [24]. We sought to identify and annotate SNVs that show significant frequency variation between asymptomatic versus severely affected COVID-19 patients. We further compared the ancestral affiliations of asymptomatic and mildly symptomatic patients to those presenting with severe disease by combining the genetic data from COVID-19 patients with 10,215 ancient and modern genomes across the globe, assessing 597,573 SNVs from the personal database of Dr. David Reich, Harvard Medical School, USA, Available online (https://reich.hms.harvard.edu/datasets, accessed on 25 March 2020). Our findings reveal discernible genomic variation between asymptomatic/mildly symptomatic and acutely affected COVID-19 patients of European ancestry.

## 2. Materials and Methods

### 2.1. Dataset

COVID-19 patient genomic data was obtained from the AncestryDNA COVID-19 host genetic study [24], through The European Genome-phenome Archive (EGA) (Accession no. EGAD00010002012) with kind permission from the AncestryDNA group (https://www.ancestry.com/dna/, last accessed on 1 August 2021). This dataset was comprised of genotyping data that included 675,370 SNVs corresponding to 11,759 healthy controls who were COVID-19 negative, and 3241 COVID-19 patients with various degrees of disease manifestations. Of the individuals included in this dataset, ~75% belonged to European ancestry [24]. The 3241 COVID-19 patient group was further categorized into asymptomatic, mild, moderate, severe and unknown based on the self-reported phenotype questionnaire (Accession no. EGAD00010002011). The methodology for data collection used to determine SARS-CoV-2 infection severity in patients has been described before [24]. To note, the patient data employed in this study came from the first wave of the pandemic. Symptoms reported by respondents include fever, shortness of breath, dry cough, nasal congestion, fatigue, headache, nausea/vomiting, diarrhoea, altered sense of taste or smell, abdominal pain, cough producing phlegm, sore throat, runny nose, chills and body ache. Based on the participant responses, we used the criteria described in Table 1 and grouped 249 individuals as asymptomatic, 283 as mild, 455 as moderate, 1907 as severe and 347 as unknown. All patients who progressed to being affected with pneumonia were included in the severe category.

### 2.2. Generation of Genomic Datasets

The COVID-19 genomic dataset from AncestryDNA was subsequently merged with one of the most recent genome datasets (v42.4.1240K_HO) obtained from the publicly available database of Dr. David Reich’s at Harvard Medical School, USA, at https://reich.hms.harvard.edu/datasets (accessed on 25 March 2020). The Reich lab data is comprised of 10,215 ancient and modern genomes from across the globe, assessing 597,573 SNVs. The final merged dataset (COVID+Ancient+Modern) comprised 133,829 SNVs that are common between the two datasets, assessing 25,198 individuals. All file conversions and manipulations were performed using EIGENSTRAT (EIG) v7.2 [25] and PLINK v1.9 [26].

**Figure 1:**
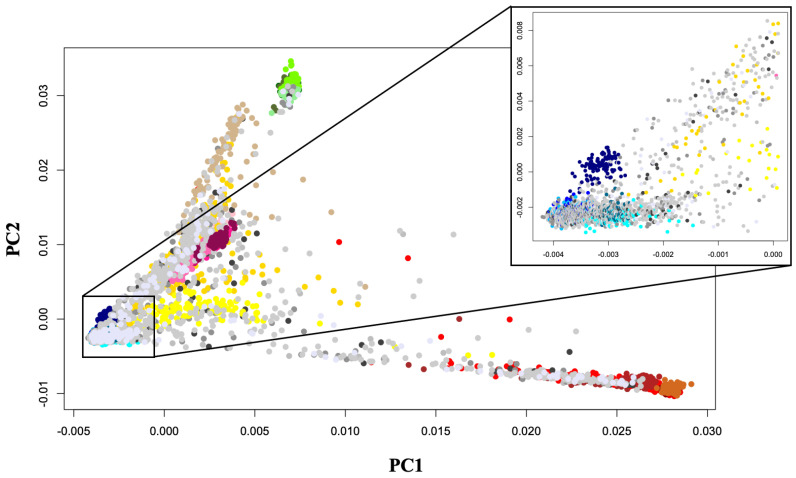
Principal Component Analysis (PCA) of COVID-19 patient genomes. PCA plot showing genetic differentiation among COVID-19 patient genomes. COVID-19 patients (asymptomatic, mild, moderate and severe) were designated in various shades of grey, and patients whose symptoms were unknown have been denoted in lavender. East Asian, European, African, South Asian and Native American populations were designated with various shades of green, blue, red, pink and yellow, respectively. To avoid cluttering, only notable populations have been plotted. We selected COVID-19 patients that cluster with individuals of European ancestry (PC1 ranging from −0.0042 to 0 and PC2 ranging from −0.0025 to 0.0067) for downstream analysis. PCA was performed in PLINK v1.9, and the top four principal components (PCs) were extracted. Top two PCs (PC1 and PC2), explaining the highest variance of the data were plotted in R v3.5.1.

Fine population structure within COVID-19 patient genomes present in COVID+Ancient+Modern dataset was delineated using Principal Component Analysis (PCA) implemented in PLINK v1.9 using --pca command. The two most informative PCs are discussed and plotted in R v3.5.1 (Figure 1). To control for population stratification and to avoid genetic structure in the sample, we only selected COVID-19 patients that cluster with individuals of European ancestry. We identified the PC coordinates for the COVID-19 patients based on a European cluster formed by CEU, FIN, GBR, IBS and ITU individuals from the 1000 Genomes Project, present in our dataset. COVID-19 patient genomes lying within the European cluster were selected (PC1 ranging from −0.0042 to 0 and PC2 ranging from −0.0025 to 0.0067) for downstream analysis (Figure 1 inset), and those outside it were removed from downstream analysis.

**Figure 2:**
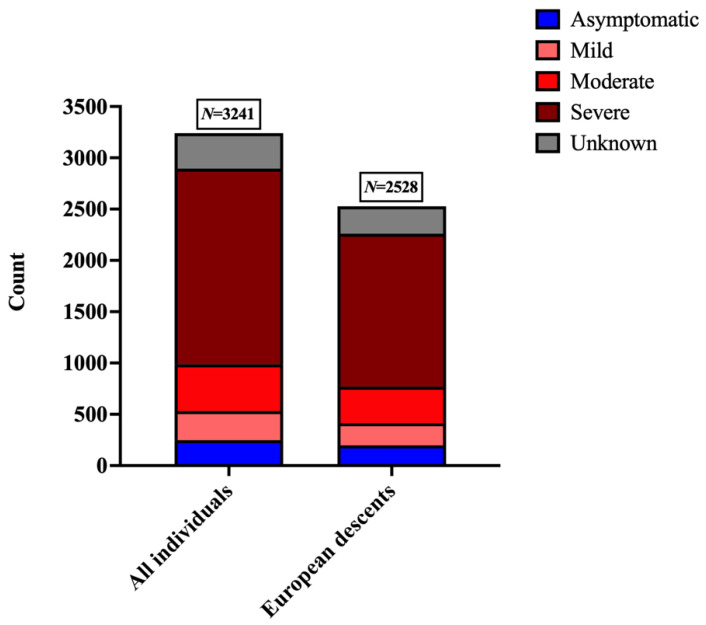
Number of samples from AncestryDNA that belong to five categories: asymptomatic, mild, moderate, severe and unknown based on self-reported severity of COVID-19. The number of COVID-19 patients in each category from the AncestryDNA dataset was compared to the number of COVID-19 patients of European descent selected by PCA for the same categories.

We selected 2528 COVID-19 patients of European descent, based on PCA, among which 197 were asymptomatic, and 217,355 and 1492 patients had mild, moderate and severe symptoms, respectively. The symptomatic status of 267 individuals was unknown (Figure 2). Data pertaining to healthy individuals were excluded from further analysis because while they were not affected with COVID-19 at the time of data collection, this does not preclude the possibility of SARS-CoV-2 infection in them at subsequent time-points. Accordingly, a new dataset was generated (COVID+Ancient+Modern_mod), comprising 10,215 ancient and modern genomes from across the globe, and 2528 COVID-19 patients of European descent (*N* = 12,743), assessing 133,829 SNVs for ancestry analysis.

### 2.3. Genome-Wide Association Analyses (GWAS)

GWAS was performed using the original AncestryDNA COVID-19 genotyping dataset (EGA Accession no. EGAD00010002012) with 675,370 SNVs to identify genetic variants that show significant frequency variation between asymptomatic versus severely infected COVID-19 patients. Accordingly, the genomes of the asymptomatic individuals (*N* = 197) (controls) were compared against those with severe disease presentation (*N* = 1492) (cases).

As quality control (QC) measures, SNVs and individuals with high levels of data missingness (>20%) were filtered out using --geno 0.2 and --mind 0.2 flags in PLINK v1.9. Further, to filter out the rare SNVs with low minor allele frequencies (MAF) that may reduce the power for detecting SNV-phenotype association, we employed a MAF threshold of 0.01. While no individual was removed during filtering, 14 and 74,787 SNVs were removed due to missing genotype data and the MAF threshold. The final dataset consisted of 1689 individuals (197 controls + 1492 cases) encompassing 600,569 SNVs.

Standard case-control-based association analyses were performed in PLINK v1.9 using --assoc command. Multiple-testing corrected *p*-values were obtained from --adjust flag alongside --assoc command. Chi-square test, implemented in PLINK --assoc command, was performed separately for all 600,569 SNVs to statistically assess their significance, and multiple-testing corrected *p*-value < 0.001 was considered significant.

Since age has been considered as an important risk factor associated with the severity of COVID-19, a separate age-adjusted GWAS was performed in PLINK v1.9 using --mh flag alongside --adjust and --assoc commands, wherein the asymptomatic and severely affected patients were divided into two groups: above and below 50 years of age, respectively.

Manhattan plots were generated in ‘qqman’ package in R v3.5.2 [27] by plotting -Log_10_ *p*-values of all assessed SNVs in both non-age-adjusted and age-adjusted GWAS outputs. Significant SNVs (multiple-testing corrected *p*-value <0.001) were annotated using SNPnexus web-based server for GRCh38/hg38 [28].

### 2.4. Population Clustering and Ancestry Determination

The genomic ancestry of 12,743 individuals, present in the COVID+Ancient+Modern_mod dataset, assessing 133,829 SNVs, was estimated using the model-based clustering algorithm ADMIXTURE v1.3 [29]. The optimum number of ancestral components (K) was determined by minimizing the cross-validation error (CVE) using a --cv flag to the admixture command line. The lowest CVE was estimated for K = 17 (Figure S1).

Further, various ancestry fractions of 2261 COVID-19 patients with known symptomatic status (asymptomatic, mild, moderate or severe) of European ancestry were compared using One-way ANOVA, implemented in GraphPad Prism v9 (https://www.graphpad.com, accessed on 1 August 2021).

We further developed several multiple linear regression models with different combinations of various European ancestry fractions obtained from ADMIXTURE, alongside demographic and healthcare information pertaining to age, gender, body mass index (BMI) and comorbidities based on the self-reported phenotype questionnaire (Accession no. EGAD00010002011) for 2261 COVID-19 patients of European descent with known symptomatic status, in order to statistically evaluate their impact on the severity of COVID-19 presentation. SARS-CoV-2 infected individuals were graded according to the degree of the clinical manifestation; for example, asymptomatic patients were graded as 1, and symptomatic patients with mild, moderate and severe disease were graded as 2, 3 and 4, respectively. Additionally, a multiple logistic regression model was developed based on the demographic and healthcare information pertaining to age (≥50 vs. <50 years), gender (Male vs. Female), body mass index (BMI) (<25 vs. ≥25) and comorbidities (Present vs. Absent), as mentioned in the self-reported phenotype questionnaire to assess which demographic and physiological factor(s) potentially augment the severity of COVID-19. Multiple regression analyses were performed in GraphPad Prism v9. Two-tailed tests were performed considering the null hypothesis of no association of ancestral, demographic and medical factors with the degree of severity of COVID-19 manifestation and *p*-value < 0.05 was considered statistically significant.

### 2.5. Ancestry Proportions among COVID-19 Patients of European Ancestry

The COVID+Ancient+Modern dataset (*N* = 25,198) was used here. The ancestry proportions of 2,261 COVID-19 patients of European descent with various degrees of disease presentation were assessed in this analysis. A total of 267 individuals with unknown symptoms were excluded. We employed *qpAdm* [30] implemented in AdmixTools v5.1 [31] to estimate ancestry proportions in the European genomes originating from a mixture of ‘reference’ populations by utilizing shared genetic drift with a set of ‘outgroup’ populations. To note, in *qpAdm*, the target and source populations are referred to as “left” populations and the reference populations are called “right” populations. Some 14 ancient genomes, namely Luxembourg_Loschbour.DG, Luxembourg_Loschbour_published.DG, Luxembourg_Loschbour, Iberia_HG (*N* = 5), Iberia_HG_lc, Iberia_HG_published, LaBrana1_published.SG, Hungary_EN_HG_Koros (*N* = 2), Hungary_EN_HG_Koros_published. SG, were grouped together as West European Hunter-Gatherers (WHGs); three ancient genomes, namely Russia_EHG, Russia_HG_Samara and Russia_HG_Karelia (*N* = 2), were grouped together as Mesolithic Hunter-Gatherers of Eastern Europe (EHGs); 11 ancient genomes, namely Kazakhstan_MLBA_Alakul_Maitan_o (*N* = 2), Kazakhstan_MLBA_OyDzhaylau_o, Russia_MLBA_Sintashta_o1 (*N* = 2), Russia_MLBA_Sintashta_o2 (*N* = 5), Russia_MLBA_Sintashta_o3, were grouped together as Ancient North Eurasians (ANEs), two ancient genomes: KK1.SG, SATP.SG, were grouped as Caucasus Hunter-Gatherers (CHGs), and Sweden_HG_Motala (*N* = 8) were renamed as Scandinavian Hunter-Gatherers (SHGs) in *qpAdm* analysis. We tried to model modern-day Europeans with different combinations of Neolithic Near-East populations such as Neolithic Iranians (Iran_GanjDareh_N), Neolithic Anatolians (Anatolia_N) and Natufians (Israel_Natufian) alongside various combinations of ancient European Hunter-Gatherer populations (WHG, EHG, CHG, ANE and SHG) as source populations as described before [32]. We inferred that COVID-19 patients of European ancestry (target population) could be best modelled as a combination of three source populations, namely ANEs, WHGs and Neolithic Iranians (Iran_GanjDareh_N) as Left (WHG, ANE, Iran_GanjDareh_N). We used a mixture of eight ancient and modern-day populations: Ust Ishim, MA1, Kostenki14, Han, Papuan, Chukchi, Karitiana, Mbuti as the ‘Right’ outgroup populations (O8).

## 3. Results

### 3.1. Genome-Wide Association Analyses (GWAS)

**Figure 3:**
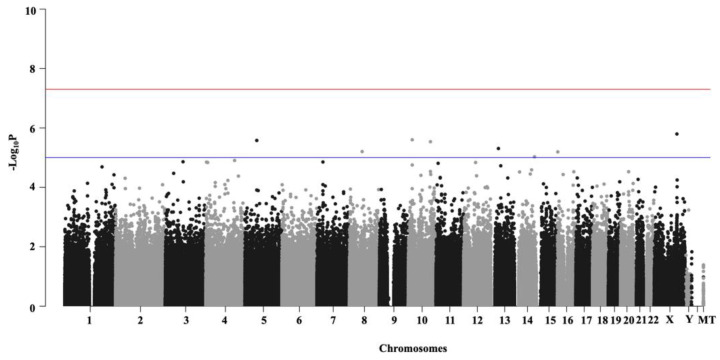
Manhattan Plot summarizing GWAS results for the age-no-adjusted cohort. *X*-axis represents chromosomes (chr 1 to chr MT). SNVs present in the chromosomes are designated with dots. Negative log-transformed (−log10) multiple-testing corrected *p*-values are plotted in *Y*-axis. 1492 COVID-19 patient genomes with severe symptoms were compared against 197 asymptomatic patient genomes. Out of 600,569 SNVs employed, 621 SNVs markers revealed highly significant variation between asymptomatic and severe cases. The SNVs with *p*-value < 0.00001 are indicated with the blue line, and those with *p*-value < 0.0000001 are indicated with the red line.

We compared the genomes of asymptomatic COVID-19 patients (*N* = 197) (controls) with that those characterized by severe disease (*N* = 1492) (cases). Out of 600,569 SNVs employed in GWAS, 621 and 615 SNVs (~0.1%) revealed significant variation (multiple-testing corrected *p*-value < 0.001) between the asymptomatic and severe categories of SARS-CoV-2 infected individuals in non-age-adjusted (Figure 3) and age-adjusted (Figure S2) association analyses, respectively. Among the significant SNVs, 612 were common between the age-adjusted and non-adjusted cohorts. Nine SNVs were uniquely present in the non-age-adjusted cohort, while three were uniquely present in the age-adjusted cohort. We annotated the 621 SNVs, identified in the non-age-adjusted cohort using SNPnexus web-based server [28].

Among the 621 appreciably distinct SNVs, 18 were found to be significantly associated (*p*-value < 0.05) with host immune system-related pathways, discerned using ‘pathway’ analysis implemented in SNPnexus. Our results indicate that genetic variants involved in pathways governing host immunity, such as innate and adaptive immune system, interferon (IFN) signalling, interleukin (IL) signalling, antigen processing by major histocompatibility complex (MHC) and cytokine signalling, showed significant variation between asymptomatic and severe COVID-19 patients (Table S1). Pathway analysis identified two SNVs, rs1042994 and rs4364309, that are significantly (*p*-values < 0.01) associated with modulating infectious diseases, hinting at likely differential cellular responses between asymptomatic and severe COVID-19 hosts to the viral onslaught. It further identified rs9427097 in *ADAR* gene, associated with mRNA editing: A to I conversion and interferon signalling. *ADAR* has been speculated to be associated with SARS-COV-2 infection [33].

Further, out of the 621 SNVs, 30 were found to be significant (*p*-value < 0.01) in ‘gwas’ implemented in SNPnexus. Among these five (rs12423247, rs7318817, rs2923084, rs17808461 and rs10077875) were associated with high body-fat related traits, such as phospholipid levels in blood plasma, high levels of high-density lipoprotein (HDL), obesity-related traits and higher BMI (Table S2). Further, significant SNVs include rs1006609, associated with cardiovascular disorders, rs4337252, associated with lung function or forced vital capacity (FVC), rs9649546, associated with mean corpuscular volume (MCV) that is, the average volume of red blood cells and white blood cell count, rs3809566, associated with platelet count, and rs10045413, associated with smoking.

**Figure 4:**
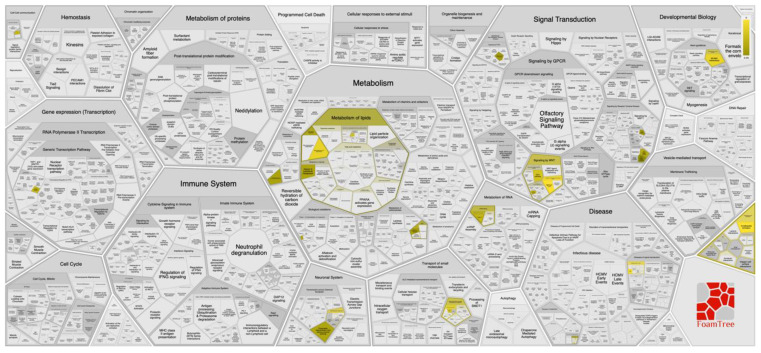
Pathway analysis of significant SNVs (*p*-value < 0.01), present on chromosome 10. Pathway map was generated using SNPnexus web-based server [28]. Significant pathways associated with these SNVs are highlighted in various shades of yellow.

We did not identify any specific genomic loci showing significant variation between the asymptomatic and severe COVID-19 patient categories (Figure 3). However, the maximum number of significantly variable SNVs (*N* = 375, multiple-testing corrected *p*-value < 0.01) were present on chromosome 10. Pathway analysis performed with these SNVs revealed them to be associated with traits such as lipid metabolism (e.g., sphingolipid metabolism) and plasma lipoprotein assembly, underscoring a putative association between obesity and the severity of COVID-19 (Figure 4) that has been suggested previously [22].

Finally, out of the three SNVs uniquely present in the age-adjusted cohort, rs8014123 is associated with thyroid carcinoma (THCA), while rs10268928 in *PTTG1IP2 Family member 2* (*PTTG1IP2*) is associated with hepatitis B virus infection (Table S2).

### 3.2. ADMIXTURE Analysis

The genomic ancestry of 12,743 individuals was estimated using the model-based clustering algorithm ADMIXTURE v1.3 [29]. The optimum number of ancestral components (K) was determined by minimizing the cross-validation error (CVE). The lowest CVE was estimated for K = 17.

**Figure 5:**
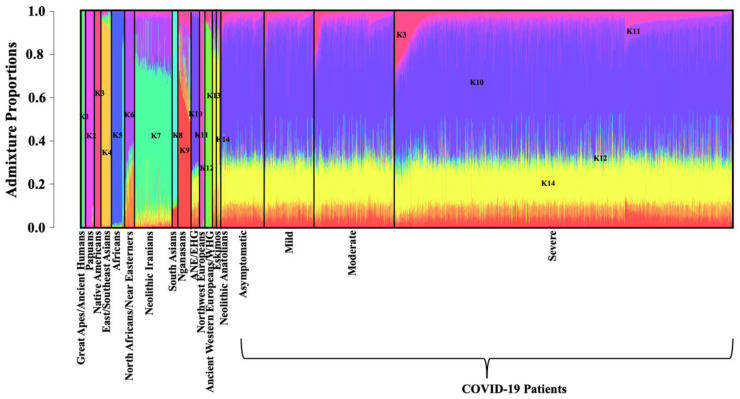
Admixture analysis of COVID-19 patients. Admixture plot showing the ancestry components of COVID-19 patients of European descent with known symptomatic status (*N* = 2261). Admixture proportions were generated through an unsupervised admixture analysis at K = 17 using ADMIXTURE v1.3 and plotted in R v3.5.1. Each individual is represented by a vertical line partitioned into coloured segments whose lengths are proportional to the contributions of the ancestral components to the genome of the individual.

At K = 17, Ancient Hominins (K = 1), Papuans (K = 2), indigenous Native American tribes (Native Americans, K = 3), East and Southeast Asians (K = 4), Sub-Saharan Africans (K5) and Ngnasans (K = 9) were assigned to distinct clusters (Figure 5). Distinct clusters of Northern Africans/Near Easterners (dominated by K6), Neolithic Iranians (K7) and South Asians (K8) were also assigned. ADMIXTURE revealed at least six clusters comprised of ancient and modern-day Europeans, namely ANE and EHG (K10), Northwest Europeans (K11), WHG (K12), Eskimos (K13), Neolithic Anatolians (K14), and Bronze and Iron Age Europeans (example, Bell Beaker and Corded ware) (Figure 5). The 2261 COVID-19 patients of European descent with various degrees of disease presentations (asymptomatic, mild, moderate and severe) depicted discernible genetic admixture among K3, K10, K11, K12 and K14 ancestry fractions (Figure 5).

**Figure 6:**
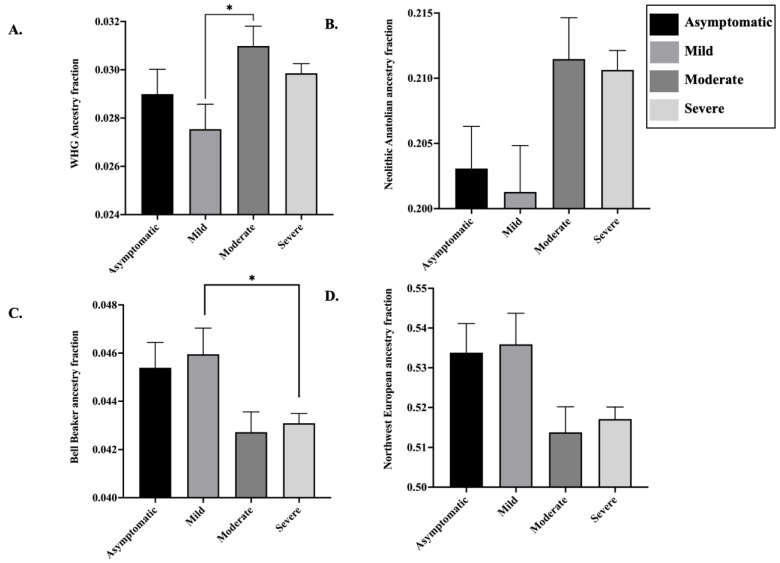
Comparison of the major admixture components among COVID-19 patients of European descent. ANOVA, followed by Tukey’s pair-wise comparison, was performed in GraphPad Prism v9, and *p*-value < 0.05 was considered significant. (**A**) Comparison of WHG (K12) ancestry. Moderately symptomatic individuals contained the highest fraction of the WHG component compared to the rest, closely followed by the severely symptomatic individuals. (**B**) Comparison of Neolithic Anatolian ancestry (K14). The lowest fraction of Neolithic Anatolian ancestry component was present among the genomes of mildly symptomatic individuals, followed by the asymptomatic people. (**C**) Comparison of Bell Beaker ancestry. Higher fractions of Bell Beaker related ancestry were present among the genomes of mildly symptomatic and asymptomatic individuals. (**D**) Comparison of Northwest European ancestry (K11). Asymptomatic and mildly symptomatic individuals depicted higher fractions of Northwest European ancestry compared to the rest. *p*-value < 0.05 is designated by ‘*’.

ANOVA revealed two distinct groups among the COVID-19 patients: while asymptomatic and mildly symptomatic individuals formed one cluster, the moderately and severely symptomatic individuals grouped separately. Although only marginally significant (One-way ANOVA, *p*-value = 0.06), moderately and severely symptomatic COVID-19 patients revealed larger WHG (K12) ancestry fractions compared to the asymptomatic and mildly symptomatic individuals, and the difference between mildly and moderately symptomatic individuals was statistically significant (Tukey’s multiple comparison, adjusted *p*-value = 0.04) (Figure 6A). Further, moderately and severely affected patients depicted significantly larger (One-way ANOVA, *p*-value = 0.04) Neolithic Anatolian ancestry fractions (K14) compared to the asymptomatic and mildly symptomatic patients (Figure 6B). However, the pair-wise difference among the four groups was not significant, likely because of the high variation in the dataset (Coefficient of variation >25%). In contrast, asymptomatic and mildly symptomatic individuals revealed significantly larger fractions of Bronze and Iron Age European ancestry, i.e., Bell Beaker ancestry fractions (One-way ANOVA, *p*-value = 0.01) and Northwest European ancestry (K11) (One-way ANOVA, *p*-value = 0.03) compared to the moderately and severely affected subjects (Figure 6C,D). The Bell Beaker ancestry fraction was significantly different between the mildly and severely symptomatic patients (Tukey’s multiple comparison, adjusted *p*-value = 0.05).

### 3.3. Multiple Regression

Multiple logistic regression analysis revealed highly significant association between the male gender and the severity of COVID-19 (Odd’s Ratio = 1.78 (95% CI: 1.39–2.28), |Z| = 4.6, *p*-value < 0.0001) and significant association between comorbidities and COVID-19 severity (Odd’s Ratio = 1.36 (95% CI: 1.05–1.75), |Z| = 2.33, *p*-value = 0.02). However, we found only marginally significant association between age and the acuteness of COVID-19 pathology (Odd’s Ratio _[Age < 50]_ = 0.79 (95% CI: 0.62–1.02), |Z| = 1.79, *p*-value = 0.07). While this might be attributed to the smaller sample size of asymptomatic versus severely symptomatic patients, it could still be biologically meaningful. We did not find any association between BMI and the severity of COVID-19 (Odd’s Ratio _[BMI < 25]_ = 0.91 (95% CI: 0.69–1.2), |Z| = 0.66, *p*-value = 0.51). We attribute the lack of association between BMI and the severity of COVID-19 to the age and gender variation in the dataset, both of which have previously been linked to BMI [34].

Congruent with the ADMIXTURE results, our multiple linear regression models revealed highly significant positive association between the severity of COVID-19 disease presentation with Neolithic Anatolian ancestry fraction (*t* = 2.921, *p*-value = 0.004) and highly significant negative association between the severity of COVID-19 presentation with Bronze and Iron Age European ancestry, i.e., Bell Beaker (*t* = −2.839, *p*-value = 0.005) and Northwest European ancestry fractions (*t* = −2.711, *p*-value = 0.006). However, the association between WHG (*t* = 1.564, *p*-value = 0.11) ancestry fraction and the severity of COVID-19 was found to be marginal. Overall, our results indicate that males with higher fractions of Neolithic Anatolian and WHG ancestry and underlying comorbidities have a significantly greater propensity towards developing severe COVID-19. In contrast, females and individuals without comorbidities who possess higher fractions of Bell Beaker and Northwest European ancestries have a discernibly lower predisposition towards developing severe COVID-19.

### 3.4. Ancestry Proportions in the European Genomes Present in COVID-19 Patient Dataset

We modelled all Europeans present in the dataset as a combination of three source populations, namely WHGs, ANEs and Neolithic Iranians, in *qpAdm* analysis (see Methods). Among the 2261 COVID-19 patients of European descent, individuals with severe symptoms contained the highest WHG ancestry proportions (24%) and the lowest ANE ancestry fractions (27.1%) (Table 2). On the contrary, asymptomatic individuals were found to have low WHG ancestry proportions (22.9%) and the highest ANE ancestry fractions (28.9%) (Table 2). Interestingly, we found a clinal variation in the ancestry proportions of the symptomatic individuals, such that patients with mild and moderate symptoms depicted WHG (22.5% and 23.5%, respectively) and ANE (28.7% and 27.9%, respectively) ancestry fractions intermediate between the asymptomatic individuals and acutely ill COVID-19 patients (Table 2).

To assess the robustness of these results, we further modelled these Europeans as a combination of three source populations, namely WHGs, EHGs and Neolithic Iranians, as performed previously [32]. We found similar clinal variation in the ancestry proportions of the symptomatic individuals, such that the patients with mild and moderate symptoms depicted WHG (19.6% and 20.9% respectively) and EHG (22.2% and 21.5% respectively) ancestry fractions intermediate between that of the asymptomatic (WHG: 20.4% and EHG: 22.2%) and severe COVID-19 patients (WHG: 21.5% and EHG: 21.0%) (Table 3).

## 4. Discussion

Genetic variability contributes to the observed disparities in many diseases, including those with complex environmental and socio-economic determinants. For example, prostate cancer is the second most prevalent cancer diagnosis and the fifth most common cause of death in men worldwide; however, it disproportionately affects men of African ancestry [35]. Similarly, cardiovascular disease is a leading health problem worldwide, but its risk is governed by individual and population-scale variation in rare/Mendelian as well as common genomic regions [36,37]. SARS-CoV-2 infection causes extensive disparities in clinical manifestation among affected patients. We employed GWAS using the AncestryDNA COVID-19 genotyping dataset corresponding to individuals of European ancestry to identify novel genetic variants that were significantly distinct between asymptomatic and severely affected COVID-19 patients. We identified 621 and 615 SNVs that revealed significant variation (multiple-testing corrected *p*-value < 0.001) between the asymptomatic and severely symptomatic patients in non-age-adjusted (Figure 3) and age-adjusted (Figure S2) cohorts, respectively. Among the significant SNVs, 612 were common between the two categories. We could map the 621 SNVs to 265 genes among which, the highest number of genes (*N* = 11) were associated with heteromeric G-protein signalling pathway, which has been linked to COVID-19 in several studies. Hameid et al. (2021) recently argued that SARS-CoV-2 could alter signalling cascades either by activating the G protein-coupled receptors (GPCRs) or by directly modulating G protein signalling [38]. Vascular inflammation, associated with COVID-19, has also been linked to GPCRs [39]. Further, *G protein subunit alpha 15* (*GNA15*), one of the 11 genes discerned here, is associated with G-protein signalling and has enhanced expression in immune cells according to The Human Protein Atlas (http://www.proteinatlas.org, last accessed on 1 August 2021) [40] and therefore might play a role in the progression of COVID-19. The second greatest number of genes (*N* = 6) were found to be associated with the Wnt signalling pathway, which has also been linked to the pathological progression of COVID-19 [41].

Consistent with the importance of the host immune system in combating coronavirus infections [42], we discerned significant differences in genetic variants involved in IFN, IL and cytokine signalling pathways between asymptomatic and severe COVID-19 patients (Table S1). All of the above-mentioned immune response pathways have been previously linked to COVID-19 [43]. Notably, the hyperproduction of proinflammatory cytokines, such as IL-1 preferentially targeting lung tissue, has been linked to necessitating ICU admission in COVID-19 patients [44]. We also identified several variants associated with obesity and blood cholesterol traits (e.g., HDL). It is noteworthy that the HDL scavenger receptor B type 1 (SR-B1) has been shown to facilitate SARS-CoV-2 cellular attachment, entry and infection and HDL promotes viral infection [45], likely exacerbating COVID-19 pathologies. Finally, obesity has emerged as one of the most important risk factors of COVID-19 [22,46] and is thought to account for doubling the risk of hospitalization [22]. We would like to emphasize here that while our findings indicate that there is a conserved set of SNVs which are independently predictive of both severe COVID-19 symptoms and risk factors, such as obesity, higher cholesterol level and habits such as smoking, these SNVs themselves might not be causative of severe COVID-19 symptoms.

We note that we did not find any association of genetic variants in *ABO*, *TYK2*, *DPP9*, *IFNAR2*, *PPP1R15A*, *LZTFL1*, and *SLC6A20* loci with the severity of COVID-19 that were reported previously [16,21,22,23,24]. This is likely attributable to the choice of individuals employed as controls in the present versus earlier GWAS. All previous association studies recruited healthy individuals who had tested negative for COVID-19 by RT-PCR in the control group or employed population controls. While such individuals did not have COVID-19 at the time of their recruitment, the possibility of subsequent SARS-CoV-2 infection and its severity in them remains unknown. As a result, these groups of healthy controls may still contain underlying genetic signatures that could make them susceptible to severe COVID-19 if infected by SARS-CoV-2 at future time points. Hence, we argue that these individuals are likely not suitable controls for predicting genetic variants associated with severe COVID-19. In contrast, in the present study, we have considered the asymptomatic COVID-19 patients as controls. The absence of perceptible known disease symptoms among the asymptomatic COVID-19 subjects makes them more valuable as controls as it is suggestive of their genetic make-up, potentially playing a crucial protective role in them from severe disease outcomes.

Our findings demonstrated stark variances in host genetic factors between asymptomatic and seriously affected SARS-CoV-2 infected patients of European ancestry, outlining differences in key pathways governing host immunity and COVID-19 comorbidity attributes.

Our ADMIXTURE analysis revealed two distinct clusters among the COVID-19 patients: one composed of the asymptomatic and mildly symptomatic individuals and the other comprising the moderately and severely symptomatic patients. We found that the asymptomatic and mildly affected individuals have significantly larger fractions of Bronze and Iron Age European and Northwest European ancestries (Figure 6C,D) and lower proportions of WHG and Neolithic Anatolian related ancestry fractions (Figure 6A,B). These results were supported by the linear regression analysis, where various European ancestry fractions were modelled alongside demographic attributes, for example, age, gender and physiological factors, such as BMI and other COVID-19 comorbidities. We discerned that the Northwest European ancestry fractions were composed of ancient Viking genomes from Sweden alongside modern-day Icelandic and British genomes. The Bronze and Iron Age European ancestry fractions largely included the ancient European Bell Beaker and Corded Ware genomes. The Bell Beaker culture spread across Western and Central Europe from 2750–2500 BC to 2200–1800 BC. This expansion of the Beaker complex to Western Europe introduced high levels of Steppe-related ancestry to the British Isles and Western Europe [47]. The Bell Beaker culture was an off-shoot of the Corded Ware complex that spread across Germany and surrounding countries ~5000 years ago and shared material culture with Steppe groups such as Yamnaya herders [30]. Notably, both Bell Beaker and Corded Ware genomes had a smaller proportion of indigenous WHG ancestry fractions [47]. Consistent with these findings, our *qpAdm* analysis revealed discernibly lower fractions of WHG related ancestry among asymptomatic and mildly symptomatic individuals compared to COVID-19 patients with severe disease (Table 2A). The asymptomatic individuals also revealed higher fractions of ANE related ancestry, which was introduced to Central and Western Europe through the Yamnaya culture (Table 2A) [30]. Repeating the *qpAdm* analysis by replacing ANEs with EHGs, who derive ~75% of their ancestry from the former [30], led to uncovering discernibly higher proportions of EHG related ancestry among the asymptomatic individuals and its lowest fractions among the seriously ill COVID-19 patients (Table 2B). Overall, our studies suggest that asymptomatic and mildly symptomatic individuals derived significantly larger proportions of their ancestry from ANEs/EHGs, which was introduced to Europe through Bell Beaker culture (Yamnaya related), the severely symptomatic COVID-19 patients possess significantly larger fractions of WHG related ancestry. It is noteworthy that the variation in ancestry or admixture fractions (~2%) between severely symptomatic and asymptomatic COVID-19 patients observed here is higher than that among several genetically heterogenous and endogamous populations across highly genetically diverse countries, such as India [48] and hence cannot be considered as minuscule.

The findings in the current study may have been influenced by the limited availability of genetic data for COVID-19 patients. Among the SARS-CoV-2 infected cohort, we sub-categorized patients as asymptomatic, mild, moderate or severe and the rest as unknown based on a self-reported questionnaire. Since the aforesaid categories were delineated solely based on the questionnaire, we cannot exclude the likelihood of miscategorization, which might have influenced our results. Further, the unavailability of the genomic data for COVID19 patients in the ICU or those who may have succumbed might cause some discrepancies in the final outcomes of our study. Finally, we note that, as in the case of previous association studies, our GWAS findings may not be exclusively ascribed to SARS-CoV-2 infections alone. However, we surmise that utilizing asymptomatic COVID-19 patients as controls improves the probability of the genetic variants identified herein to being associated with the degree of COVID-19 manifestation specifically, as these are significantly different between the severely symptomatic and asymptomatic COVID-19 subjects.

Overall, our findings elucidate the striking genetic differences between asymptomatic and severely affected patients infected with SARS-CoV-2 infection. Expanding this approach to include whole-genome sequencing data and increasing the power of analysis by employing a large number of individuals in various categories of SARS-CoV-2 infection severity accentuates the potency for uncovering novel genetic variants that may be associated with severe COVID-19 in future, thereby likely identifying cellular pathways that may be targeted to develop or improve therapeutics. Further, using a population genetics driven approach such as ours in diverse ancestries will provide the opportunity to interrogate population and ancestry specific genetic factors that may govern susceptibility to severe COVID-19 and may uncover clinically actionable and more efficacious population-specific drug targets.

## 5. Conclusions

The current study shines a light on the striking differences in the genetic architecture between asymptomatic and severely affected COVID-19 patients of European descent. While asymptomatic individuals contain significantly larger ANE/EHG ancestral fractions, patients with severe clinical manifestations possess dominant WHG fractions. Host pathways governing immunity such as innate and adaptive immune system, IFN, IL, cytokine pathways, antigen processing and SNVs related to comorbidity attributes, such as obesity-related traits, cardiovascular disorders, lung function and smoking, were discerned to vary significantly between severe and asymptomatic COVID-19 patients.

## Figures and Tables

**Table 1:** Classification of COVID-19 patients based on self-reported questionnaire (EGA Accession no. EGAD00010002011).

Category	Criteria
Asymptomatic	individuals reported experiencing no perceptible symptoms
Mild	(a) All symptoms were reported as mild (b) One of the symptoms was reported as moderate, and the rest were mild
Moderate	(a) Two or more symptoms were reported as moderate (b) One of the symptoms was reported as severe,* and the rest are listed as moderate
Severe	(a) Two or more severe symptoms reported (b) Pneumonia
Unknown	symptom responses are unavailable

* Excludes Pneumonia. All pneumonia patients were considered as severe.

**Table 2:** West European Hunter-Gatherers (WHGs), Ancestral North European (ANE) and Neolithic Iranian ancestry fractions among COVID-19 patients of European descents.

Patient Category	WHG Ancestry	ANE Ancestry	Neolithic Iranian Ancestry
Asymptomatic	22.9%	28.9%	48.2%
Mild	22.5%	28.7%	48.7%
Moderate	23.5%	27.9%	48.6%
Severe	24.0%	27.1%	48.9%

**Table 3:** West European Hunter-Gatherers (WHGs), European Hunter-Gatherer (EHG) and Neolithic Iranian ancestry fractions among COVID-19 patients of European descents.

Patient Category	WHG Ancestry	EHG Ancestry	Neolithic Iranian Ancestry
Asymptomatic	20.4%	22.2%	57.4%
Mild	19.6%	22.2%	58.3%
Moderate	20.9%	21.5%	57.7%
Severe	21.5%	21.0%	57.5%

## Data Availability

The data employed in this study can be obtained from the AncestryDNA COVID-19 host genetic study [24], through The European Genome-phenome Archive (EGA: https://ega-archive.org, accessed on 18 February 2021) (Accession no. EGAD00010002012).

## Supplementary Materials

The following are available online at https://www.mdpi.com/article/10.3390/life11090921/s1, Figure S1: Figure S1_CVE_plot.tiff, Figure S2: Figure S2_Manhattan Plot300.png, Table S1: Table_S1_Immune_system_related_SNVs_identified_in_GWAS.xlsx, Table S2: Table_S2_Significant_pathways_identified_in_GWAS.xlsx.
