# Supplementary figures and images for: Genomic and Ancestral Variation Underlies the Severity of COVID-19 Clinical Manifestation in Individuals of European Descent
Source: Life (Basel). 2021 Sep 5;11(9):921. doi: 10.3390/life11090921 (PMC8470085; doi:10.3390/life11090921)

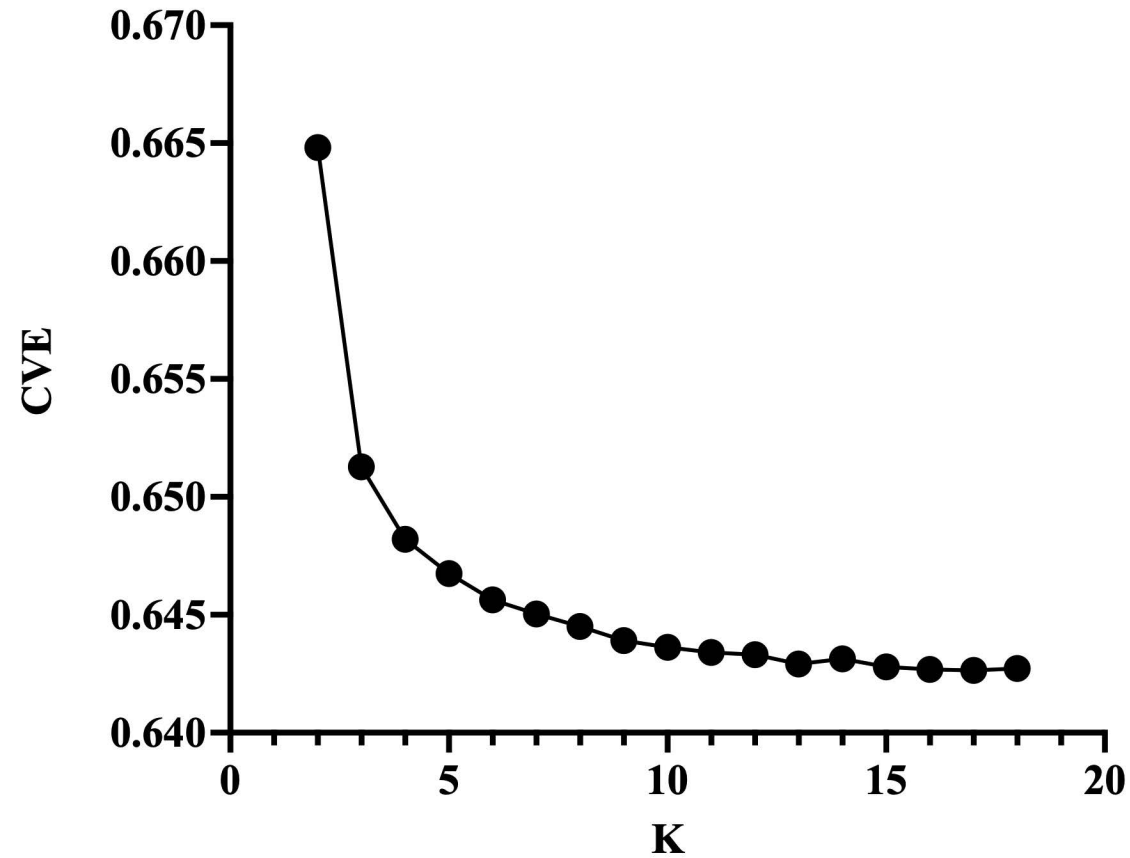

Supplement: Supplementary file 1 [file life-11-00921-s001.zip › Figure S1_CVE_plot.pdf]

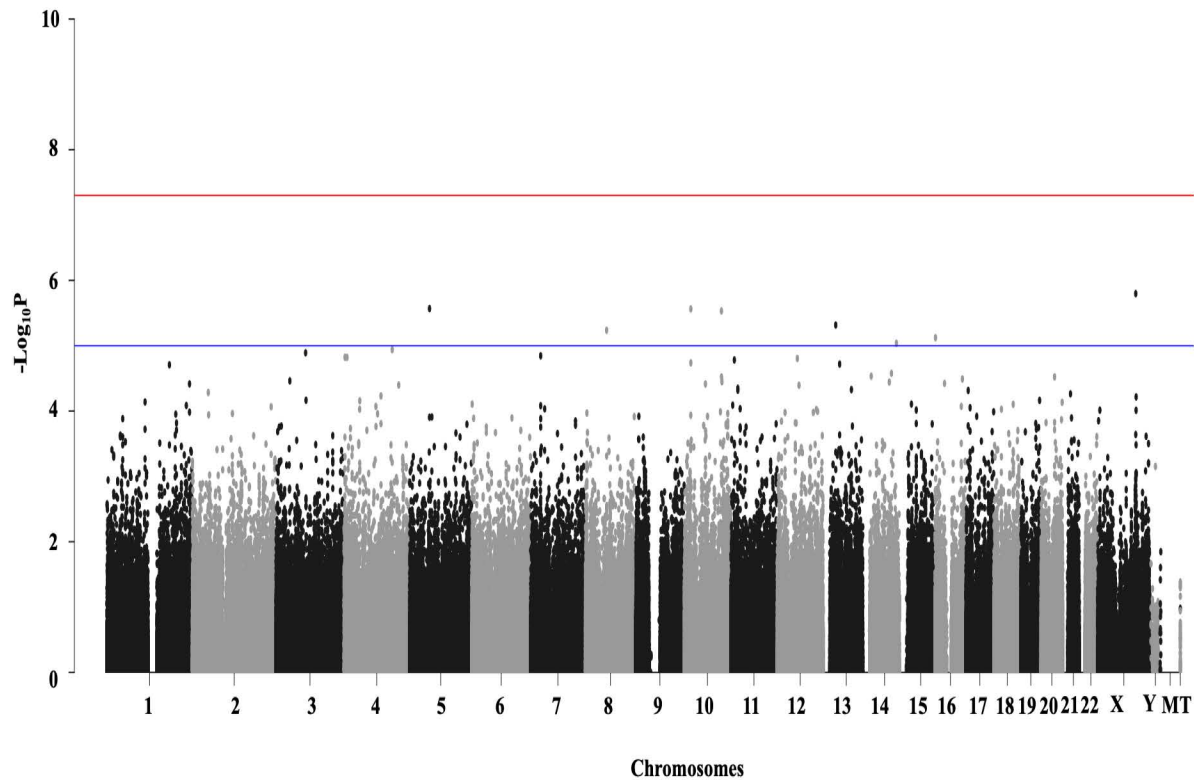

Supplement: Supplementary file 1 [file life-11-00921-s001.zip › Figure S2_Manhattan Plot300.pdf]
